# Identification of the key aroma-active compounds in wolfberry (*Lycium barbarum* L.) pulp

**DOI:** 10.1016/j.fochx.2025.103191

**Published:** 2025-10-23

**Authors:** Zhifeng Zhang, Zehao Li, Gang Fan, Lulu Zhang, JingNan Ren, Kangning Wu, Youli Ma

**Affiliations:** a College of Food Science and Technology, Huazhong Agricultural University, Wuhan, China; b Ningxia Huaxinda Health Technology Co., Ltd., Yinchuan, China; c College of Food Science and Technology, Henan University of Technology, Henan, China

**Keywords:** Wolfberry pulp, Aroma-active compounds, GC × GC-TOFMS, GC–MS-O, HS-GC-IMS

## Abstract

Identifying the key aroma-active compounds in wolfberry pulp is essential for ensuring product quality. In this study, the key odorants in fresh wolfberry pulp (FWP), dried wolfberry pulp (DWP) and mixed wolfberry pulp (MWP) were systematically characterized using sensory evaluation, comprehensive two-dimensional gas chromatography and time-of-flight mass spectrometry (GC × GC-TOFMS), gas chromatography-mass spectrometer-olfactometry (GC–MS-O), and headspace-gas chromatography-ion mobility spectrometry (HS-GC-IMS). A total of 58 odorants were detected across the three samples, exhibiting flavor dilution (FD) factors ranging from 1 to 243, while 33 odorants showed odor activity values (OAVs) greater than 1. Data analysis identified hexanal, (*E*)-2-hexenal, geranylacetone, 2-undecanone, 6-methyl-5-hepten-2-one, 1-hexanol, 4-hexen-1-ol, 2-hexen-1-ol, 2-pentylfuran, eremophilene, and β-selinene as the key aroma-active compounds in FWP. Furthermore, HS-GC-IMS provided distinctive fingerprint profiles that allowed clear differentiation of 84 odorants among FWP, DWP, and MWP. These findings establish a theoretical basis for the quality control and improvement of wolfberry pulp products.

## Highlights

• Characteristic aroma components of wolfberry pulps were profiled.
• Sensory analysis revealed clearly distinct aroma profiles between fresh and dried wolfberry pulps.
• The key aroma-active compounds were identified by GC × GC-TOFMS, GC–MS-O, AEDA and OAVs.
• Fingerprint profiles obtained by GC-IMS can distinguish the types of wolfberry pulps.

## Graphical abstract

**Figure:**
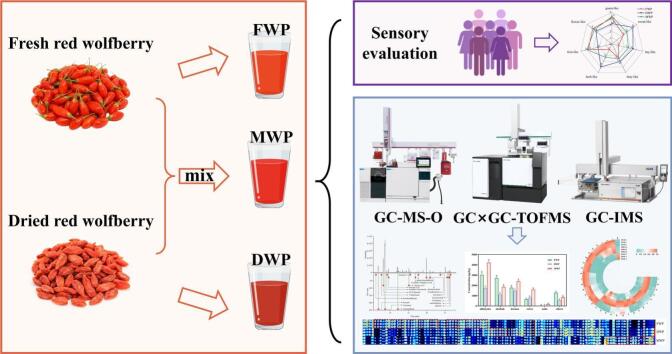

## 1 Introduction

Wolfberry (*Lycium barbarum* L.), a small shrub belonging to the family Solanaceae, produces bright orange berries typically 1–2 cm in length (Yao et al., 2018). In addition to its widespread use as a food ingredient, wolfberry has long been utilized in traditional herbal medicine for its reputed ability to nourish the liver and kidneys. Recent studies have demonstrated that wolfberry is rich in various bioactive substances, including polysaccharides, carotenoids, polyphenols, and phytosterols, which exhibit antioxidant, anti-inflammatory, and various other health-promoting activities, such as cytoprotective, retinoprotective, immunostimulatory, and antithrombogenic effects (Huang et al., 2019; Vidović et al., 2022).

Owing to its high nutritional and medicinal value, the market demand for wolfberry has been steadily increasing, accompanied by a continuous expansion in its cultivation scale. However, due to its soft texture, wolfberry is highly prone to mechanical damage and spoilage during storage and transportation. To extend its shelf life and satisfy market needs, wolfberry has been processed into a variety of products, such as juices, wines, teas, and concentrated tablets (Liu et al., 2019; Ren et al., 2018). In recent years, wolfberry pulp, which retains the fruit's nutritional properties relatively well, has emerged as a novel and increasingly popular product in the food industry (Wang et al., 2024).

The aroma profile of wolfberry pulp is a critical determinant of its quality and strongly influences consumer preference. However, most existing studies have primarily relied on gas chromatography–mass spectrometry (GC–MS) and headspace-gas chromatography-ion mobility spectrometry (HS-GC-IMS) to analyze aroma components (Wang et al., 2024; Zhao et al., 2023; Zhou et al., 2023). These techniques present certain limitations, including low sensitivity, insufficient separation capacity, and high peak overlap, which hinder the comprehensive characterization and decoding of key aroma-active compounds in wolfberry pulp. In contrast, comprehensive two-dimensional gas chromatography and time-of-flight mass spectrometry (GC × GC-TOFMS) exhibits superior performance in separating complex odor compounds compared with conventional GC–MS, providing a more detailed understanding of volatile compositions (Wen et al., 2023). Moreover, combining GC–MS with olfactometry enables both the identification and sensory evaluation of odor compounds, thereby facilitating the effective detection of key aroma-active constituents (Baba et al., 2017).

In addition, due to the delicate and highly perishable nature of fresh wolfberry fruit, dried wolfberries are commonly used as raw material for the industrial production of wolfberry pulp. However, the drying process can trigger a series of chemical reactions, such as the Maillard reaction and oxidative degradation. These transformations may result in the loss or modification of odorants, thereby significantly affecting the aroma quality of the final pulp products (Huang et al., 2025; Zheng et al., 2025a). Therefore, elucidating the differences in aroma compounds between fresh and dried wolfberry pulps is essential for understanding and improving product quality.

In this study, sensory evaluation was first conducted to assess the differences in overall aroma characteristics among fresh wolfberry pulp (FWP), dried wolfberry pulp (DWP), and mixed wolfberry pulp (MWP). Subsequently, the aroma compositions of FWP, DWP, and MWP were comprehensively characterized using GC × GC-TOFMS and gas chromatography–mass spectrometry-olfactometry (GC–MS-O). The key aroma-active compounds were then identified based on flavor dilution (FD) factors and odor activity values (OAVs). Finally, fingerprint information obtained from HS-GC-IMS was employed to further elucidate differences in odorants among the three types of wolfberry pulp. Overall, the findings of this study provide a theoretical foundation for a deeper understanding of the aroma characteristics of wolfberry pulps and support data-driven approaches to quality control and product improvement.

## 2 Material and methods

### 2.1 Chemicals

Compounds hexanal, (*E*)-2-hexenal, 4-heptenal, octanal, nonanal, β-cyclocitral, acetone, acetoin, 1-pentanol, 1-hexanol, ethyl acetate, hexyl acetate, ethyl pentanoate, 2-ethylfuran, d-limonene, and 2-pentylfuran were purchased from Shanghai Yuanye Bio-Technology Co., Ltd. (Shanghai, China). The compound 2-methyl-3-heptanone was obtained from Shanghai Aladdin Biochemical Technology Co., Ltd. (Shanghai, China). The n-alkanes (C_7_-C_40_) were procured from Sigma-Aldrich (St. Louis, MO, USA).

### 2.2 Preparation of wolfberry pulps

Fresh and dried red wolfberries were collected from Ningxia, China, in 2024, and all samples used for pulp preparation were commercially ripe. The wolfberries were first rinsed with distilled water and then juiced using a MJ-ZZ12W7–002 juicer (Midea Co., Ltd., China). The resulting mixture was filtered through a 100-mesh sieve, homogenized at 10000 ×*g* for 5 min using an FSH-2 A homogenizer (Changzhou Yineng Experimental Apparatus Factory, China), and subsequently pasteurized at 95 °C for 30 min to obtain FWP and DWP, respectively. For MWP, fresh and dried red wolfberries were combined at a 1:1 ratio (*w*/w), and then sequentially subjected to cleaning, juicing, filtration, homogenization, and sterilization to prepare the final product. The total soluble solids content of all pulp samples was adjusted to 16–18°Brix with distilled water prior to analysis. The prepared pulps were then cooled to room temperature (25 ± 0.5 °C) and immediately subjected to aroma compound analysis. Each sample (2 g) was analyzed in triplicate, with three injections per replicate.

### 2.3 Sensory evaluation of wolfberry pulps

A sensory evaluation was conducted by a panel of 15 trained assessors, comprising 8 men and 7 women aged 22–32 years. Each panelist evaluated three randomly coded samples (FWP, DWP, and MWP), with three replicates per sample. For each evaluation, 10 g of wolfberry pulp was transferred into a glass vial for assessment. The panelists described the main odor attributes as “green-like”, “honey-like”, “hay-like”, “fatty-like”, “herb-like”, “fruit-like”, and “flower-like”. The intensity of each attribute was rated on a structured 0–5 scale, where 0 represented no perception, 3 indicated moderate intensity, and 5 denoted strong perception (He et al., 2024; Tang et al., 2024).

### 2.4 Extraction of aroma compounds

Solid-phase microextraction (SPME) using a divinylbenzene/carboxen/polydimethylsiloxane fiber (50/30 μm, Supelco, Bellefonte, PA, USA) was employed to extract aroma compounds from wolfberry pulps. Each 20 mL headspace vial contained 2 g of wolfberry pulp and 1 μL of 2-methyl-3-heptanone. The samples were heated at 60 °C for 20 min to achieve equilibration, after which volatile compounds were adsorbed onto the fiber at 50 °C for 35 min. The fiber was then desorbed into the GC injection port for 3 min to release the volatiles. All extractions were performed in triplicate for each sample.

### 2.5 Identification of aroma compounds using GC × GC-TOFMS and GC–MS-O

Aroma compounds in FWP, DWP, and MWP were analyzed using an Agilent 7890B gas chromatograph coupled with an EI-0620 time-of-flight mass spectrometer and an SSM1820 solid-state thermal modulator. Volatile compounds were adsorbed onto the SPME fiber at 50 °C for 35 min and subsequently thermally desorbed at 250 °C for 3 min. Separation was performed using two columns: an Agilent DB-WAX column (30 m × 0.25 mm × 0.25 μm) and a DB-17MS column (1.0 m × 0.15 mm × 0.15 μm), with an SV column used as the modulation column. The GC oven program was set as follows: initial temperature of 40 °C (held for 5 min), increased at 3 °C/min to 250 °C. Helium was used as the carrier gas at a constant flow rate of 1.4 mL/min. The solid-state thermal modulator was operated with an inlet temperature of 30 °C, an outlet temperature of 120 °C, and a modulation period of 5 s. The MS conditions were as follows: ion source temperature, 230 °C; transfer line temperature, 240 °C; electron ionization (EI) energy, 70 eV; and scan range, *m*/*z* 47–350. Compound identification was carried out using Canvas 1.0.0.25117 (J&X Technology, China) in combination with the NIST 20 database.

Additionally, aroma compounds in FWP, DWP, and MWP were analyzed using an Agilent 7890 A gas chromatograph coupled with a 5975C series mass spectrometer and an olfactometric detection port (Gerstel ODP2, Germany). A nonpolar HP-5MS column (60 m × 250 μm × 0.25 μm) was employed, with chromatographic conditions identical to those used in the GC × GC-TOFMS analysis. The effluent was split equally between the olfactory port and the MS detector at a ratio of 1:1. Odor evaluation was conducted by three trained panelists, and each volatile compound's characteristics were confirmed by at least two evaluators (Jia et al., 2021). Compound identification was performed qualitatively using the NIST 17 database.

### 2.6 Aroma extract dilution analysis (AEDA)

FD factors of odorants were determined using SPME-AEDA combined with GC–MS-O. During the analysis, the split ratio was gradually increased from 1:3 to 1:243. The sniffing procedure was terminated when the evaluators could no longer perceive any odor, and the FD factor was recorded as the highest dilution at which the odor was still detectable (Jia et al., 2021).

### 2.7 Quantification of aroma compounds

Aroma compounds in wolfberry pulps were quantified using an internal standard calibration method. The concentrations of odorants were determined from calibration curves established by correlating GC–MS peak areas with the relative ratios of the target analytes to the internal standard, 2-methyl-3-heptanone (Han et al., 2021; Hao et al., 2023).

### 2.8 OAVs analysis

The OAV method has widely been used to evaluate the contribution of individual aroma compounds to the overall sensory profile. OAV is defined as the ratio of the concentration of an aroma compound to its corresponding odor threshold (OT). In this study, the OTs in water were obtained from previously published literature (Bonneau et al., 2016; Gao et al., 2025; Huang, Huang, et al., 2023; Kim & Chung, 2009; Lee et al., 2008; Li et al., 2007; Niu et al., 2019; Oguz et al., 2021; Tamura et al., 2020; Tang et al., 2012; Yang et al., 2019).

### 2.9 HS-GC-IMS analysis

HS-GC-IMS analysis of aroma compounds in wolfberry pulps was implemented as previously described by Jia et al. (2024). A GC-IMS system (FlavorSpec®, G.A.S, Dortmund, Germany) equipped with an MXT-WAX column (30 m × 0.53 mm i.d., 1.0 μm film thickness, Düren, Germany) and an autosampler (CTC-PAG, Zwingen, Switzerland) was used. Briefly, 2 g of wolfberry pulp was placed in a 20 mL headspace vial and equilibrated at 60 °C for 20 min. Subsequently, 500 μL of the headspace gas was automatically injected into the IMS inlet (85 °C, splitless mode). Nitrogen (99.99 % purity) served as the carrier gas, with the flow rate programmed as follows: 2 mL/min (0–2 min), 10 mL/min (2–10 min), 100 mL/min (10–20 min), and held at 100 mL/min (20–50 min). The drift tube temperature was maintained at 45 °C, with a drift gas flow rate of 75 mL/min. Retention indexes (RIs) of odorants were calculated using n-ketones (C_4_-C_9_) as external references, and compound identification was achieved by comparing their RIs and drift times with those of standards in the HS-GC-IMS library.

### 2.10 Statistical analysis

Variance analysis was performed using SPSS 19.0 software (SPSS Inc., USA). Statistical significance was determined at *P* < 0.05 using one-way analysis of variance (ANOVA) followed by Tukey's b test to assess differences among samples. Heatmap visualizations were generated using the Chiplot online platform (https://www.chiplot.online/).

## 3 Results and discussion

### 3.1 Sensory evaluation of wolfberry pulps

**Fig. 1:**
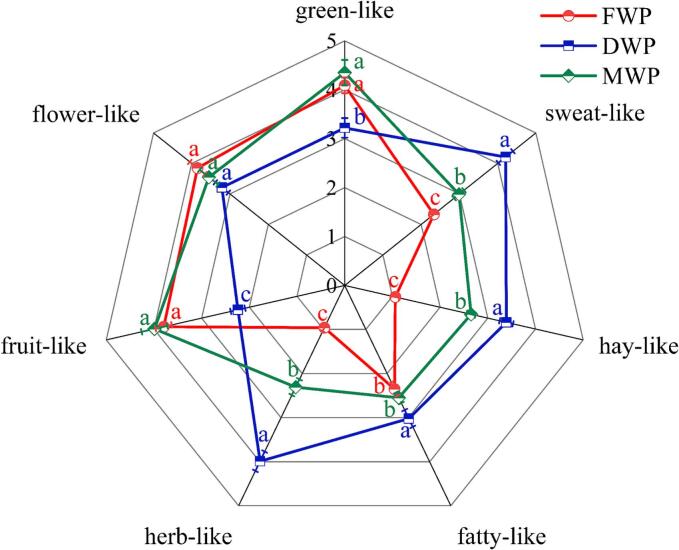
Sensory evaluation of wolfberry pulps. Lowercase letters (a, b, and c) on the same odor attribute represent significant differences at *P* < 0.05. FWP, fresh wolfberry pulp. DWP, dried wolfberry pulp. MWP, mixed wolfberry pulp.

Odor attributes and descriptions of FWP, DWP, and MWP were displayed in Fig. 1. Based on the sensory panel's evaluation of overall aroma, FWP exhibited a more intense and preferred aroma profile. No significant differences were observed among FWP, DWP, and MWP in terms of flower-like aroma characteristics (*P* > 0.05). However, significant variations were detected in sweat-like, herb-like, and hay-like attributes. Specifically, both FWP and MWP displayed similarly fruit-like, flower-like, and green-like notes. In contrast, DWP was primarily characterized by sweat-like, hay-like, fatty-like, and herb-like odors, with markedly lower intensities of fruit-like and green-like aromas compared to FWP and MWP (*P* < 0.05). These findings were consistent with those of Zheng, Oellig, Vetter, et al., 2025, Zheng, Oellig, Zhu, et al., 2025), who also reported that hay-like and fatty-like attributes were dominant aroma characteristics of dried goji berries based on sensory analysis.

### 3.2 Identification of aroma compounds in FWP, DWP and MWP

In this study, GC × GC-TOFMS and GC–MS-O analyses were employed to characterize the aroma compounds in FWP, DWP, and MWP. As shown in Table 1, a total of 61 odorants were identified, comprising 16 aldehydes, 16 alcohols, 12 ketones, 5 esters, 3 acids, and 9 others. Among these, 58 odorants in FWP, DWP, and MWP were perceptible by GC–MS-O, with FD factors ranging from 1 to 243. Previous studies on wolfberry aroma have mainly focused on fermented wolfberry juices (Qi et al., 2021; Zhao et al., 2023), black wolfberries (Zhou et al., 2023), goji berries, and goji berry pulps (Lu et al., 2017; Peng et al., 2024; Wang et al., 2024). Notably, several compounds, including aldehydes (nos. ***2*** and ***16***), alcohols (nos. ***18***, ***19***, ***21***, ***24***, and ***31***), ketones (nos. ***34***, ***35***, and ***41***), ester (no. ***49***), acid (no. ***50***), others (nos. ***54*** and ***57***), were detected for the first time in wolfberry pulps.

**Table 1:** The retention index (RI), odor notes, and flavor dilution (FD) factors of aroma compounds in fresh wolfberry pulp (FWP), dried wolfberry pulp (DWP), and mixed wolfberry pulp (MWP) based on GC × GC-TOFMS and GC–MS-O analyses.

Group	No	Identified odorants	RI	Retention time		Odor notes	FD factors			Identification
				1D (min)	2D (s)		FWP	DWP	MWP	
aldehydes	1	butanal	–	2.866	4.852	pungent, cocoa, musty	3	3	1	MS, O
	2	isovaleraldehyde	–	3.450	0.885	chocolate, fatty	9	n.s.	27	MS, O
	3	hexanal	1066	7.866	1.464	fresh, green, fatty	243	27	243	MS, RI, O
	4	2-hexenal	1185	12.783	1.426	sweet, almond, bitter	1	n.s.	n.s.	MS, RI, O
	5	(*E*)-2-hexenal	1200	13.450	1.348	green, banana	81	3	9	MS, RI, O
	6	4-heptenal	1226	14.616	1.527	oily, fatty, dairy	1	1	1	MS, RI, O
	7	octanal	1275	16.783	1.938	waxy, citrus	3	1	1	MS, RI, O
	8	nonanal	1381	21.450	1.994	rose, fresh, orris	9	1	27	MS, RI, O
	9	furfural	1442	24.033	0.763	sweet, woody, bready	3	3	81	MS, RI, O
	10	benzaldehyde	1496	26.283	1.054	bitter, almond	9	3	9	MS, RI, O
	11	2-nonenal	1517	27.116	1.637	green, cucumber	1	1	3	MS, RI, O
	12	β-cyclocitral	1596	30.283	1.865	rose, sweet, green	27	27	27	MS, RI, O
	13	β-homocyclocitral	1596	30.283	2.016	oily, berry, orris	9	1	1	MS, RI, O
	14	phenylacetaldehyde	1618	31.116	1.039	floral, hyacinth	9	3	9	MS, RI, O
	15	safranal	1620	31.120	1.647	woody, medicinal, spicy	9	27	27	MS, RI, O
	16	3-ethylbenzaldehyde	1679	33.450	1.277	sweet, almond	9	3	3	MS, RI, O
alcohols	17	2-methyl-1-propanol	1086	8.616	0.706	fresh, alcoholic, leather	1	1	1	MS, RI, O
	18	1-pentent-3-ol	1152	11.366	0.723	green, radish	3	3	1	MS, RI, O
	19	3-pentanol	1202	13.533	0.778	fusel, alcoholic	1	3	9	MS, RI, O
	20	1-pentanol	1245	15.450	0.789	oily, sweet	1	3	1	MS, RI, O
	21	2-pentenol	1312	18.450	0.710	aldehydic, cherry	1	1	1	MS, RI, O
	22	2-heptanol	1316	18.616	0.992	mushroomy, oily	1	3	3	MS, RI, O
	23	1-hexanol	1350	20.116	0.837	resinous, floral, green	243	27	27	MS, RI, O
	24	4-hexen-1-ol	1375	21.199	0.795	green, vegetable, tomato	27	n.s.	1	MS, RI, O
	25	2-hexen-1-ol	1400	22.283	0.840	fresh, green, fruity	81	3	3	MS, RI, O
	26	1-heptanol	1450	24.366	0.898	musty, leafy	3	n.s.	9	MS, RI, O
	27	2-ethylhexanol	1484	25.783	0.967	fresh, floral	1	1	3	MS, RI, O
	28	linalool	1542	28.116	1.074	woody, green	3	1	3	MS, RI, O
	29	1-octanol	1552	28.533	0.950	rose, mushroomy	9	1	1	MS, RI, O
	30	2-nonen-1-ol	1703	34.366	0.928	violet, melon	3	3	1	MS, RI, O
	31	benzyl alcohol	1854	39.699	0.639	phenolic, balsamic	1	n.s.	1	MS, RI, O
	32	phenylethyl alcohol	1888	40.866	0.732	rose, floral	3	n.s.	3	MS, RI, O
ketones	33	acetone	–	2.366	4.631	ethereal, apple, pear	3	3	3	MS, O
	34	3-penten-2-one	1110	9.533	1.171	fruity	1	1	1	MS, RI, O
	35	4-methyl-2-hexanone	1168	12.033	1.699	fruity, spicy	9	n.s.	9	MS, RI, O
	36	3-octanone	1241	15.283	2.024	herbal, lavender	9	n.s.	3	MS, RI, O
	37	acetoin	1265	16.366	0.704	buttery, creamy	1	9	3	MS, RI, O
	38	2-octanone	1271	16.616	1.839	cheesy, mushroomy	3	n.s.	9	MS, RI, O
	39	2,2,6-trimethylcyclohexanone	1295	17.699	2.255	honey, cistus	9	1	3	MS, RI, O
	40	6-methyl-5-hepten-2-one	1323	18.950	1.559	citrus, green, musty	27	9	3	MS, RI, O
	41	benzoin	1496	26.279	1.446	balsamic, vanilla	n.s.	n.s.	3	MS, RI, O
	42	2-undecanone	1586	29.866	2.035	fruity, fatty	27	81	27	MS, RI, O
	43	geranylacetone	1837	39.116	1.809	fresh, green, fruity	27	1	27	MS, RI, O
	44	β-ionone	1916	41.783	1.797	woody, orris, berry	9	3	9	MS, RI, O
esters	45	ethyl acetate	–	3.116	0.748	sweet, weedy	1	3	9	MS, O
	46	butyl acetate	1059	7.618	1.459	fruity, candy	1	1	1	MS, RI, O
	47	hexyl acetate	1262	16.1998	2.029	green, apple	3	1	3	MS, RI, O
	48	methyl salicylate	1745	35.866	1.200	wintergreen, minty	1	n.s.	9	MS, RI, O
	49	ethyl pentanoate	1257	43.227	1.134	pineapple, green	1	3	1	MS, RI, O
acids	50	formic acid	–	3.783	4.608	pungent, vinegar	1	1	1	MS, O
	51	acetic acid	1440	23.950	0.481	pungent, vinegar	3	3	81	MS, RI, O
	52	hexanoic acid	1834	39.033	0.566	fatty, cheesy	3	n.s.	9	MS, RI, O
others	53	2-ethylfuran	–	4.116	1.020	beany, cocoa, bready	9	3	3	MS, O
	54	ethylbenzene	1108	9.450	1.819	aromatic odor	1	3	3	MS, RI, O
	55	d-limonene	1182	12.616	2.839	citrus, orange, fresh	1	3	27	MS, RI, O
	56	2-pentylfuran	1219	14.283	1.963	beany, fruity	243	81	243	MS, RI, O
	57	eremophilene	1688	33.783	3.188	leather, woody	27	n.s.	n.s.	MS, RI
	58	β-selinene	1692	33.949	3.132	woody, herbal	27	n.s.	n.s.	MS, RI
	59	valencene	1701	34.283	3.118	citrus, green	9	n.s.	n.s.	MS, RI, O
	60	4-hydroxy-3-methoxystyrene	2164	49.699	0.729	spicy, clove	3	3	9	MS, RI, O
	61	4-vinylphenol	2359	55.366	0.497	phenolic, medicinal, spicy	3	n.s.	3	MS, RI

n.s. represents not smelled. MS indicates that compounds are identified by MS spectra (NIST 20). O represents odor attributes perceived at the sniffing port. FD factors are defined as the highest spilt ratio at which the odorant could be perceived by SPME dilution.

#### 3.2.1 Aldehydes

The FD factor is recognized as a critical parameter for the preliminary screening of aroma-active compounds. Table 1 presented the FD factors of odorants identified in FWP, DWP, and MWP. Aldehydes were predominant volatile compounds in all wolfberry pulps. Specifically, 16 aldehydes exhibited the highest FD factors, including hexanal (243 vs 27 vs 243), (*E*)-2-hexenal (81 vs 3 vs 9), nonanal (9 vs 1 vs 27), furfural (3 vs 3 vs 81), β-cyclocitral (27 vs 27 vs 27), and safranal (9 vs 27 vs 27) in FWP, DWP, and MWP, respectively. These results implied that the aroma compounds in DWP underwent a certain degree of loss, likely resulting from the co-evaporation of volatiles with water during the drying process. Hexanal, which imparts a characteristic green odor note, has previously been detected in fresh and dried wolfberries as well as in various wolfberry-derived products (Liu et al., 2022; Niu et al., 2019; Wang et al., 2024; Zheng et al., 2025a; Zheng et al., 2025b).

Other saturated straight-chain aldehydes, such as octanal and nonanal, were also detected in FWP, DWP, and MWP. Octanal imparts characteristic “fruity” and “lemon” odors. Although aldehydes are generally associated with “fruity” and “floral” aromas, elevated concentrations, particularly of those with carbon chain lengths exceeding C6, can produce distinct “fatty” odor notes. The enzymatic oxidation of oleic acid in fruits, catalyzed by lipoxygenase, may lead to the formation of nonanal. This compound contributes “grassy”, “floral”, and “fatty” sensory impressions. Previous studies have reported that nonanal possessed a relatively low OT, thereby exerting a more pronounced influence on the overall aroma profile of juice (Wang et al., 2024).

Furfural, a common volatile compound characterized by “sweet,” “woody,” and “bready” aromas, is typically formed through the caramelization or Maillard reaction (Huang, Chen, et al., 2023). Owing to the relatively high sugar content of wolfberries, elevated temperatures during drying or pulp processing may facilitate furfural generation (Chen et al., 2015). Previous studies have identified furfural as a key contributor to the sweet-like odor of wolfberry pulps (Peng et al., 2024; Wang et al., 2024; Zhao et al., 2023). Besides, MWP integrated the aroma characteristics of both fresh and dried wolfberry pulps. Thus, furfural and other odorants originating from DWP may synergistically interact with volatile components from FWP, creating a distinctive aroma profile. Such interactions may enhance the prominence of furfural in MWP, potentially explaining its higher FD factor.

In addition, odorants such as β-cyclocitral, characterized by “rose,” “sweet,” and “green” notes, and safranal, exhibiting “woody,” “medicinal,” and “spicy” aromas, have been previously reported in wolfberry pulps (Meng et al., 2019; Peng et al., 2024). Overall, based on FD factors, the aldehyde-derived aroma profiles of FWP were comparable to those of MWP, whereas DWP exhibited markedly weaker odor intensities.

#### 3.2.2 Alcohols

Apart from aldehydes, alcohols were also identified as major aroma constituents in wolfberry pulps. These compounds are typically derived from the oxidative degradation of fatty acids and are mainly associated with “green,” “fresh,” “sweet,” “floral,” and “mushroom-like” odors (Xu et al., 2024). An increase in alcohol content generally contributes to the enhancement of desirable aroma characteristics (Peng et al., 2024). Several alcohols, including 2-methyl-1-propanol, 1-pentanol, 2-heptanol, 1-hexanol, 2-hexen-1-ol, 1-heptanol, 2-ethylhexanol, linalool, 1-octanol, 2-nonen-1-ol, and phenylethyl alcohol, have been detected in wolfberries and their derived products (Peng et al., 2024; Qi et al., 2021; Wang et al., 2024; Zhao et al., 2023; Zhou et al., 2023). Screening of the alcohols revealed that the C6 alcohols 1-hexanol (FD = 243 vs 27 vs 27; resinous, floral, green) and 2-hexen-1-ol (FD = 81 vs 3 vs 3; fresh, green, fruity) were potent odorants in FWP, DWP, and MWP, respectively. Lipoxygenase and hydroperoxide lyase catalyze the conversion of linoleic and linolenic acids into C6 aldehydes, which are subsequently reduced by alcohol dehydrogenase to form the corresponding C6 alcohols. Additionally, 1-octanol (FD = 9 vs 1 vs 1; rose-like, mushroomy) and 3-pentanol (FD = 1 vs 3 vs 9; fusel-like, alcoholic) were also detected in FWP, DWP, and MWP. Furthermore, 4-hexen-1-ol (FD = 27 vs 1; green, vegetable-like, tomato-like), 1-heptanol (FD = 3 vs 9; musty, leafy), benzyl alcohol (FD = 1 vs 1; phenolic, balsamic), and phenylethyl alcohol (FD = 3 vs 3; rose-like, floral) were newly identified as potent odorants in FWP and MWP, respectively.

#### 3.2.3 Ketones

Ketones are typically associated with “green,” “fruity,” “herbal,” and “woody” odors in wolfberry pulps. As shown in Table 1, the highest FD factors in FWP, DWP, and MWP were observed for 2-undecanone (27 vs 81 vs 27), geranylacetone (27 vs 1 vs 27), and 6-methyl-5-hepten-2-one (27 vs 9 vs 3). Among these, 2-undecanone and geranylacetone, responsible for “green” and “fruit” notes, have been identified as major volatiles in wolfberries (Meng et al., 2019). Also, 6-methyl-5-hepten-2-one, produced through the oxidative cleavage of carotenoids, is recognized as a key volatile in wolfberries (Peng et al., 2024; Qi et al., 2021). Other important odorants detected in FWP, DWP, and MWP included acetoin (FD = 1 vs 9 vs 3; buttery, creamy), 2,2,6-trimethylcyclohexanone (FD = 9 vs 1 vs 3; honey-like, cistus-like), and β-ionone (FD = 9 vs 3 vs 9; woody, orris-like, berry-like), all of which have previously been reported as dominant volatiles in wolfberry pulps (Lu et al., 2017; Qi et al., 2021). Notably, 4-methyl-2-hexanone (FD = 9 vs 9; fruity, spicy), 3-octanone (FD = 9 vs 3; herbal, lavender-like), and 2-octanone (FD = 3 vs 9; cheesy, mushroomy) were exclusively detected through olfactory analysis in FWP and MWP. These results suggested that certain aroma-active ketones may be partially lost during the drying process of DWP.

#### 3.2.4 Esters

Esters, formed through the condensation of organic acids and alcohols, play a pivotal role in shaping the sensory attributes of wolfberry pulps by enhancing sweetness, fruitiness, and floral notes while mitigating sourness (Peng et al., 2024). Among the esters identified in the samples, ethyl acetate exhibited relatively high FD factors (1 vs 3 vs 9 in FWP, DWP, and MWP, respectively). This compound has been recognized as a key aroma contributor in wolfberry pulps, imparting characteristic “fruity,” “sweet,” and “weedy” notes (Wang et al., 2024). What's more, butyl acetate, hexyl acetate, and methyl salicylate have also been identified as characteristic volatile esters contributing to the overall aroma of wolfberry pulps (Lu et al., 2017; Wang et al., 2024; Zhou et al., 2023).

#### 3.2.5 Acids

Three acids, formic acid, acetic acid, and hexanoic acid, were detected in wolfberry pulps. Among them, acetic acid showed the highest FD factors (3 vs 3 vs 81 in FWP, DWP, and MWP, respectively), indicating that it was likely the most abundant acid. Previous studies have similarly identified acetic acid as the predominant aroma compound in fresh red goji berries (Zheng et al., 2025a), dried red goji berries (Zheng et al., 2025b), and Ningxia goji berries (Lu et al., 2017). This predominance may be associated with its formation via the phosphogluconate pathway or citric acid metabolism (Qi et al., 2021).

#### 3.2.6 Others

Many fruits exhibit distinctive fragrances primarily due to the presence of terpenoids. Wolfberry pulps contain various terpenoids, including d-limonene, valencene, β-selinene, and eremophilene (Peng et al., 2024). Among these, d-limonene, characterized by a “citrus, orange, and fresh” odor, was detected in FWP, DWP, and MWP with FD factors ranging from 1 to 27. In contrast, valencene (FD = 9), β-selinene (FD = 27), and eremophilene (FD = 27) were exclusively detected in FWP.

In addition, furans contribute “beany”, “cocoa”, “bready”, and “fruity” notes to wolfberry pulps. Notably, 2-ethylfuran (FD = 9 vs 3 vs 3) and 2-pentylfuran (FD = 243 vs 81 vs 243) were detected in FWP, DWP, and MWP, respectively. An earlier study identified 2-pentylfuran and 2-hexyltetrahydrofuran, which imparted “beany” and “fruity” aromas, as characteristic volatile compounds in Ningxia goji berries (Lu et al., 2017).

Furthermore, several volatile phenolic compounds were identified in FWP, DWP, and MWP, including 4-hydroxy-3-methoxystyrene (FD = 3 vs 3 vs 9; spicy, clove-like) and 4-vinylphenol (FD = 3 vs n.s. vs 3; phenolic, medicinal, spicy). Recent studies have also reported 4-hydroxy-3-methoxystyrene as an important odorant in wolfberry pulps (Wang et al., 2024).

### 3.3 Differences of the concentrations and OAVs of aroma compounds in FWP, DWP, and MWP

**Fig. 2:**
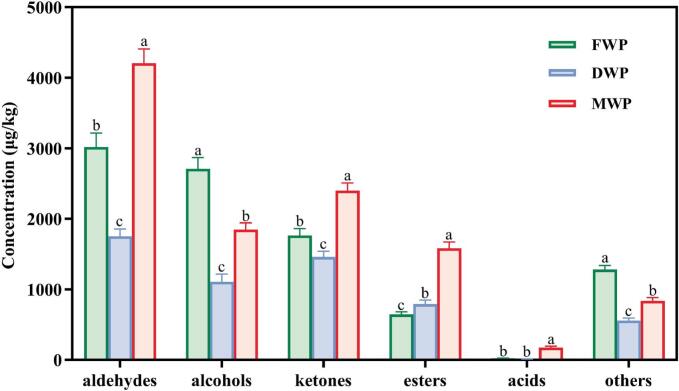
The total concentration of aroma compounds in FWP, DWP, and MWP. Lowercase letters (a, b and c) represent significant differences in the concentration of odorants at *P* < 0.05. FWP, fresh wolfberry pulp. DWP, dried wolfberry pulp. MWP, mixed wolfberry pulp.

Concentration differences of aroma compounds in FWP, DWP, and MWP were illustrated in Fig. 2. Aldehydes (3017.89 μg/kg) were the most abundant odorants in FWP, followed by alcohols (2708.17 μg/kg), ketones (1764.18 μg/kg), others (1281.71 μg/kg), esters (646.25 μg/kg), and acids (21.69 μg/kg). More specifically, aldehydes accounted for 31.97 %, 30.88 %, and 38.08 % of the total odorants in FWP, DWP, and MWP, respectively. Compared with FWP, although the concentration of esters in DWP increased by 22.16 % (*P*<0.05), significant reductions were observed in aldehydes, alcohols, ketones, others, and total odorants by 41.91 %, 59.10 %, 17.31 %, 56.45 %, and 39.86 %, respectively (*P*<0.05). Conversely, MWP exhibited significant increases in aldehydes, ketones, esters, acids, and total odorants by 39.36 %, 36.11 %, 144.89 %, 700.34 %, and 17.01 %, respectively, compared with FWP (*P*<0.05). These results suggested that during the drying process, a portion of the aroma components in wolfberries was lost; however, some characteristic volatiles remained. When dried wolfberries were blended with fresh ones to produce MWP, the aroma components from both sources can complement each other, resulting in a more complex aroma profile. Moreover, during the mixing process, chemical reactions or synergistic interactions between the constituents of dried and fresh wolfberries may contribute to the formation of new aroma compounds or the enhancement of existing aromatic characteristics.

**Fig. 3:**
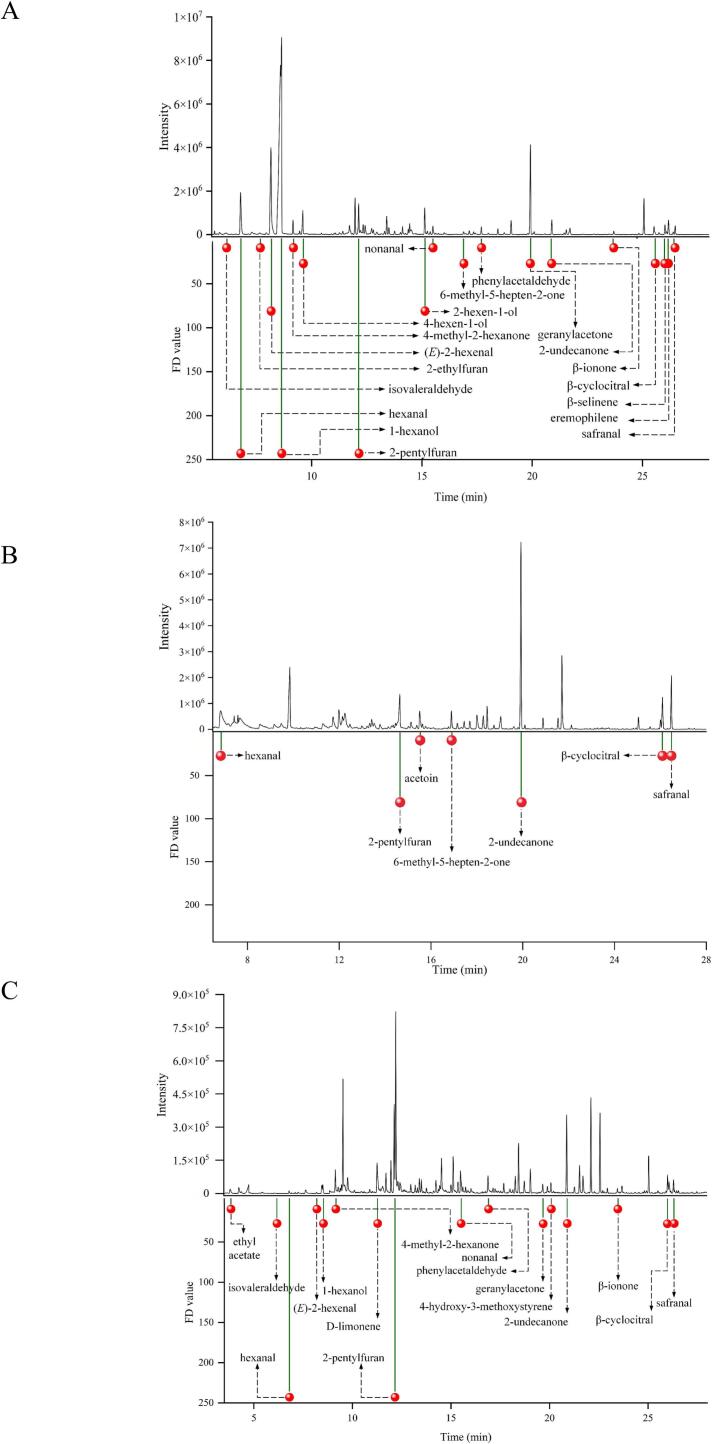
GC–MS chromatograms of key aroma-active compounds with both FD factors ≥9 and OAVs ≥1 labeled in FWP (A), DWP (B), and MWP (C). FD factors, flavor dilution factors. OAVs, odor activity values. FWP, fresh wolfberry pulp. DWP, dried wolfberry pulp. MWP, mixed wolfberry pulp.

Although the concentration of odorants in wolfberry pulps provides the foundation for understanding their aroma composition, it alone is insufficient for effectively screening and identifying aroma-active compounds. The OAV, as a crucial complementary indicator, enables a more accurate evaluation of the actual contribution of individual odorants to the overall aroma profile (Hao et al., 2023). As shown in Table 2, a total of 33 aroma-active compounds with OAVs ≥1 were identified, most of which exhibited correspondingly high FD factors. Among them, butanal (OAV = 304.4 vs 170.6 vs 232.8), isovaleraldehyde (OAV = 531.4 vs 586.9 vs 979.1), hexanal (OAV = 280.6 vs 64.1 vs 238.5), (*E*)-2-hexenal (OAV = 594.1 vs 98.1 vs 482.1), 4-heptenal (OAV =453.6 vs 398.6 vs 762.2), nonanal (OAV = 113.7 vs 43.0 vs 173.0), and safranal (OAV =106.7 vs 176.4 vs 205.0) had the high OAVs in FWP, DWP, and MWP, respectively. Most aldehydes are formed from unsaturated fatty acids through α-oxidation, β-oxidation, or lipoxygenase pathway (Xu et al., 2024). In wolfberries, linoleic acid, oleic acid, and linolenic acid serve as the main precursor fatty acids. Specifically, hexanal and (*E*)-2-hexenal originate from linoleic acid, nonanal is derived from oleic acid, and (*E*)-2-heptenal is produced from linolenic acid via oxidative metabolism (Wang et al., 2024; Zheng et al., 2025a; Zheng et al., 2025b). Besides, acetone (OAV =50.8 vs 49.7 vs 81.7), 2-undecanone (OAV = 7.3 vs 22.3 vs 23.2), and β-ionone (OAV =499.7 vs 126.1 vs 222.3) exhibited relatively high OAVs across the three types of wolfberry pulp. The formation of ketones can be attributed to the degradation of amino acids, fatty acids, or carotenoids, as well as carbohydrate metabolism. In particular, 2-undecanone likely arises from fatty acid degradation, whereas β-ionone may be formed through carotenoid breakdown. Moreover, 2-ethylfuran (OAV = 49.9 vs 29.7 vs 29.6) and 2-pentylfuran (OAV = 22.5 vs 23.9 vs 39.3) also exhibited relatively high OAVs in FWP, DWP, and MWP, respectively. By integrating both FD factors and OAVs, 19, 7, and 16 aroma-active compounds with FD factors ≥9 and OAVs ≥1 were identified in FWP, DWP, and MWP, respectively (Fig. 3). The key aroma-active compounds in FWP included hexanal, (*E*)-2-hexenal, geranylacetone, 2-undecanone, 6-methyl-5-hepten-2-one, 1-hexanol, 4-hexen-1-ol, 2-hexen-1-ol, 2-pentylfuran, eremophilene, β-selinene, and so on. These findings were consistent with those of Lu et al. (2017), who identified hexanal, (*E*)-2-hexenal, nonanal, 1-hexanol, 1-octen-3-ol, hexyl acetate, methyl salicylate, limonene, linalool, β-cyclocitral, and 2-pentylfuran as the primary aroma volatiles in Ningxia goji berries based on odorant screening via FD factors and OAVs.

**Table 2:** Comparison of OAVs of aroma compounds in FWP, DWP, and MWP.

Group	No a	Identified odorants	Concentration (μg/kg)			OTs (μg/kg)	OAVs		
			FWP	DWP	MWP		FRWP	DRWP	MRWP
aldehydes	1	butanal	153.88 ± 4.34^a^	86.61 ± 5.22^c^	116.38 ± 4.74^b^	0.5	304.4	170.6	232.8
	2	isovaleraldehyde	594.31 ± 11.22^b^	645.55 ± 52.71^b^	1076.78 ± 43.25^a^	1.1	531.4	586.9	979.1
	3	hexanal	1262.85 ± 51.61^a^	288.57 ± 23.56^b^	1073.12 ± 43.78^c^	4.5	280.6	64.1	238.5
	4	2-hexenal	143.35 ± 8.79^a^	4.12 ± 0.26^c^	38.80 ± 1.58^b^	30	4.7	<1	1.3
	5	(*E*)-2-hexenal	205.37 ± 12.60^a^	33.35 ± 2.72^c^	163.93 ± 6.69^b^	0.34	594.1	98.1	482.1
	6	4-heptenal	13.93 ± 0.98^b^	12.16 ± 0.74^b^	22.87 ± 0.93^a^	0.03	453.6	398.6	762.2
	7	octanal	17.75 ± 1.09^a^	7.96 ± 0.65^b^	17.83 ± 0.72^a^	0.7	24.9	11.4	25.5
	8	nonanal	115.74 ± 6.91^b^	42.98 ± 3.51^c^	172.98 ± 7.05^a^	1	113.7	43.0	173.0
	11	2-nonenal	11.25 ± 0.69^b^	10.33 ± 0.84^b^	22.97 ± 0.94^a^	1.1	10.1	9.4	20.9
	12	β-cyclocitral	77.06 ± 4.74^c^	105.82 ± 8.64^b^	162.68 ± 6.64^a^	5	15.2	21.2	32.5
	14	phenylacetaldehyde	13.88 ± 0.85^a^	3.65 ± 0.30^c^	10.77 ± 0.44^b^	4	3.4	<1	2.7
	15	safranal	75.95 ± 4.66^c^	123.38 ± 9.97^b^	143.47 ± 5.85^a^	0.7	106.7	176.4	205.0
alcohols	18	1-penten-3-ol	361.68 ± 22.18^b^	308.19 ± 23.44^c^	449.62 ± 18.32^a^	400	<1	<1	1.12
	20	1-pentanol	135.25 ± 7.98^b^	77.52 ± 4.44^c^	163.11 ± 6.71^a^	150.2	<1	<1	1.09
	23	1-hexanol	1311.38 ± 80.44^a^	348.14 ± 28.43^c^	665.64 ± 27.13^b^	500	2.58	<1	1.33
	24	4-hexen-1-ol	114.69 ± 6.99^a^	n.d.	2.59 ± 0.10^b^	0.1	1128.46	<1	25.95
	25	2-hexen-1-ol	493.19 ± 30.25^a^	2.20 ± 0.18^b^	3.20 ± 0.13^b^	232	2.09	<1	<1
ketones	33	acetone	1014.04 ± 23.51^b^	993.80 ± 40.52^b^	1653.53 ± 44.10^a^	20	50.8	49.7	81.7
	35	4-methyl-2-hexanone	119.33 ± 4.07^a^	n.d.	62.45 ± 2.55^b^	0.81	148.2	<1	77.1
	37	acetoin	12.28 ± 0.75^c^	83.09 ± 6.78^b^	122.46 ± 4.99^a^	14	<1	5.9	8.7
	40	6-methyl-5-hepten-2-one	189.38 ± 11.62^a^	94.29 ± 7.13^c^	132.23 ± 5.39^b^	68	2.7	1.4	1.9
	42	2-undecanone	40.90 ± 2.51^b^	122.42 ± 10.00^a^	127.48 ± 5.18^a^	5.5	7.3	22.3	23.2
	43	geranylacetone	155.19 ± 9.52^a^	4.00 ± 0.33^c^	57.54 ± 2.35^b^	60	2.5	<1	1.0
	44	β-ionone	52.84 ± 3.24^a^	13.32 ± 0.83^c^	23.12 ± 0.94^b^	0.104	499.7	126.1	222.3
esters	45	ethyl acetate	578.87 ± 23.63^c^	705.71 ± 52.35^b^	1394.06 ± 56.89^a^	500	1.2	1.4	2.8
	47	hexyl acetate	52.60 ± 3.23^b^	38.07 ± 2.37^c^	126.24 ± 4.74^a^	100	<1	<1	1.3
	49	ethyl pentanoate	11.56 ± 0.71^c^	44.07 ± 2.73^a^	15.15 ± 0.66^b^	30	<1	1.4	<1
others	53	2-ethylfuran	399.22 ± 16.27^a^	243.31 ± 16.66^b^	236.71 ± 9.57^b^	8	49.9	29.7	29.6
	55	d-limonene	20.55 ± 1.26^c^	68.52 ± 4.21^b^	143.93 ± 5.87^a^	34	<1	1.98	4.23
	56	2-pentylfuran	132.94 ± 8.13^b^	138.98 ± 6.14^b^	227.99 ± 9.29^a^	5.8	22.5	23.9	39.3
	57	eremophilene	306.88 ± 18.63	n.d.	n.d.	105	2.87	<1	<1
	58	β-selinene	216.60 ± 13.29	n.d.	n.d.	100	2.13	<1	<1
	60	4-hydroxy-3-methoxystyrene	68.32 ± 4.19^b^	44.81 ± 2.68^c^	104.01 ± 4.24^a^	12	5.60	3.67	8.67

a The number of compounds is given in Table 1. * Lowercase letters (a, b, and c) represent significant differences at *P* < 0.05. n.d., not detected. OAVs, odor activity values. OTs, odor thresholds. FWP, fresh wolfberry pulp. DWP, dried wolfberry pulp. MWP, mixed wolfberry pulp.

**Fig. 4:**
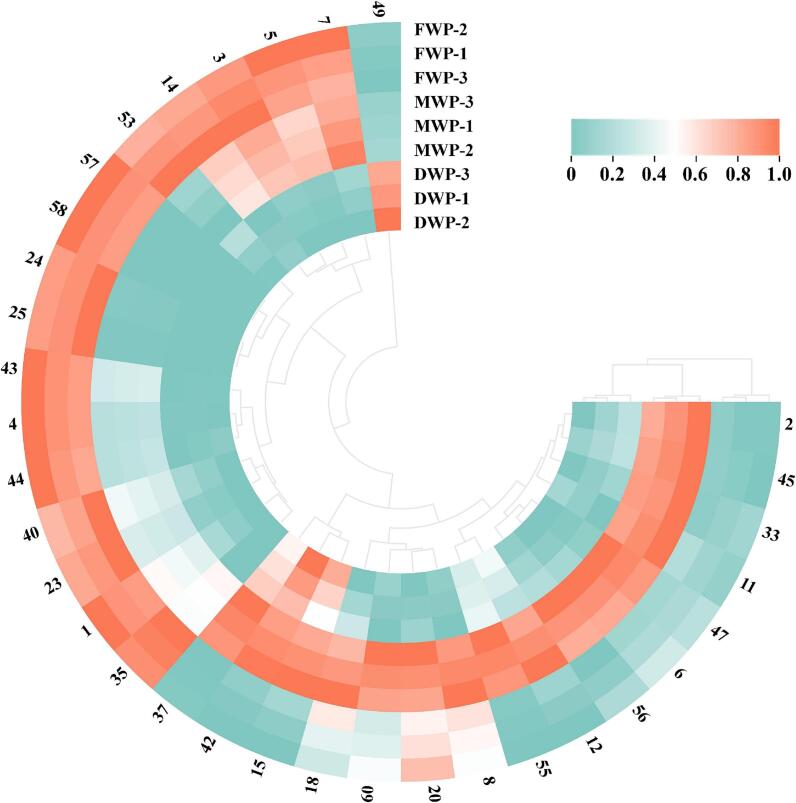
Cluster heat map of aroma-active compounds (OAVs ≥1) in FWP, DWP, and MWP. OAVs, odor activity values. FWP, fresh wolfberry pulp. DWP, dried wolfberry pulp. MWP, mixed wolfberry pulp.

Furthermore, the cluster heat map (Fig. 4) revealed distinct variations in aroma-active compounds among FWP, DWP, and MWP. Compared with FWP, the majority of aroma-active compounds (nos. ***1***, ***3***, ***4***, ***5***, ***7***, ***8***, ***14***, ***23***, ***24***, ***25***, ***35***, ***40***, ***43***, ***44***, ***53***, ***57***, ***58***) exhibited reduced intensities in DWP, while only a few odorants (nos. ***15***, ***37***, ***42***, ***49***) showed an increase. In contrast, relative to both FWP and DWP, several odorants (nos. ***2, 6, 8, 11, 12, 18, 20, 33, 45, 47, 55, 56, 60***) in MWP displayed enhanced abundance. Notably, twelve aroma-active compounds (nos. ***1, 4, 23, 24, 25, 35, 40, 43, 44, 53, 57, 58***) were identified as characteristic aroma-active compounds of FWP, distinguishing it from DWP and MWP.

### 3.4 Differences of aroma compounds in FWP, DWP, and MWP using HS-GC-IMS

**Fig. 5:**
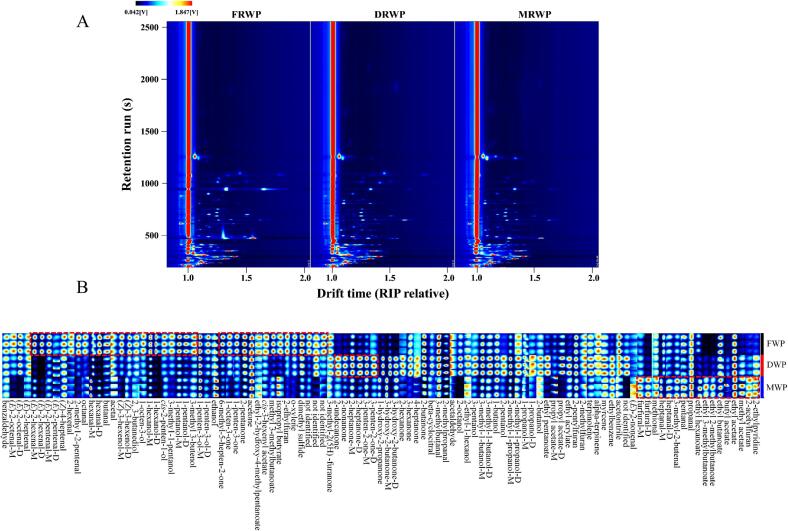
Top-view plot (A) and gallery plot (B) based on the signal intensity of aroma compounds in FWP, DWP, and MWP via HS-GC-IMS. FWP, fresh wolfberry pulp. DWP, dried wolfberry pulp. MWP, mixed wolfberry pulp.

A total of 84 odorants (nos. ***A1***-***A84***, detailed in Table 3) were detected in wolfberry pulps using HS-GC-IMS, comprising 21 aldehydes, 20 alcohols, 15 ketones, 15 esters, and 13 others. The top-view plot showed that these odorants were distributed within a retention time range of 200–1500 s (Fig. 5A). Notably, the gallery plot (Fig. 5B) revealed clear distinctions among FWP, DWP, and MWP, with the red signal intensity of each compound being positively correlated with its concentration. In FWP, dimethyl sulfide, o-xylene, 2-ethylfuran, methyl 3-methylbutanoate, *cis*-3-hexenyl acetate, 2-pentanone, 1-penten-3-one, 1-octen-3-one, 6-methyl-5-hepten-2-one, 3-methyl 3-butenol, 1-pentanol, 3-methyl-1-pentanol, *cis*-2-penten-1-ol, 1-hexanol, 1-octen-3-ol, 2, 3-butanediol, (*Z*)-3-hexenol, butanal, hexanal, octanal, 2-methyl-2-pentenal, 2-hexenal, (*E*)-2-pentenal, (*E*)-2-hexenal were identified as characteristic aroma compounds. These findings were consistent with those obtained from cluster heat map analysis of aroma-active compounds. Interestingly, except for 2-ethylfuran, 6-methyl-5-hepten-2-one, 1-pentanol, 1-hexanol, butanal, hexanal, octanal, 2-hexenal, and (*E*)-2-hexenal, the remaining compounds were exclusively detected by HS-GC-IMS and not by GC × GC-TOFMS. This observation aligned with previous studies demonstrating that HS-GC-IMS exhibited superior sensitivity in detecting low-molecular-weight volatile compounds (Jia et al., 2024). In DWP, 3-penten-2-one, 2-heptanone, 2-nonanone, 2-undecanone, 2-hexanone, and 4-heptanone were identified as characteristic aroma compounds, while pentanal, 3-methyl-2-butenal, heptanal, methional, furfural, ethyl 2-methylbutanoate, ethyl 3-methylbutanoate, ethyl hexanoate, 2-ethylpyridine, 2-acetylfuran, and methyl acetate were characteristic of MWP. Importantly, both HS-GC-IMS and GC × GC-TOFMS consistently indicated that furfural was the predominant aroma compounds in MWP, highlighting its key role in defining the mixed pulp's aroma characteristics.

**Table 3:** The aroma compounds identified in FWP, DWP, and MWP via HS-GC-IMS.

No.	Compounds a	Odor notes	RI b	Rt [*sec*] c	Dt [RIPrel] d	Comment
aldehydes						
A1	acetaldehyde	green, slight fruity	764.8	212.395	1.0227	
A2	propanal	pungent, green grassy	815.9	237.507	1.14097	
A3	2-methylpropanal	banana, melon, nutty	825.4	242.459	1.28327	
A4	butanal	pungent, fruity, green leaf	888.9	278.535	1.28049	
A5	3-methylbutanal	chocolate, fat	929	304.001	1.39969	
A6	pentanal	green grassy, faint banana, pungent	1005.5	361.652	1.42279	
A7–1	hexanal	fresh, green, fat, fruity	1101.7	478.543	1.30563	monomer
A7–2	hexanal	fresh, green, fat, fruity	1102	479.074	1.56168	dimer
A8–1	(*E*)-2-pentenal	potato, peas	1150	564.612	1.10783	monomer
A8–2	(*E*)-2-pentenal	potato, peas	1150.2	565.143	1.35508	dimer
A9	2-methyl-2-pentenal	aldehydes, soil, garlic	1167.8	600.208	1.15948	
A10–1	heptanal	fresh, aldehyde, fatty	1197.7	659.713	1.36388	monomer
A10–2	heptanal	fresh, aldehyde, fatty	1197.7	659.713	1.69245	dimer
A11	3-methyl-2-butenal	fruity	1216.3	685.214	1.09465	
A12	2-hexenal	almonds, fruity, apples	1217.1	686.277	1.18476	
A13–1	(*E*)-2-hexenal	green, banana, fat	1232.4	708.06	1.18256	monomer
A13–2	(*E*)-2-hexenal	green, banana, fat	1232.8	708.591	1.51113	dimer
A14	octanal	aldehyde, waxy, citrus, orange	1302.1	816.734	1.44207	
A15	(*E*)-2-heptenal	spicy, green vegetables	1332.8	872.779	1.25511	
A16–1	(*E*)-2-octenal	fatty, green herbal	1431.3	1079.88	1.35037	monomer
A16–2	(*E*)-2-octenal	fatty, green herbal	1431.3	1079.88	1.81054	dimer
A17	methional	onion, meat, fruity	1476.6	1190.88	1.08977	
A18–1	furfural	sweet, woody, bready	1496.8	1243.958	1.08977	monomer
A18–2	furfural	sweet, woody, bready	1497.2	1245.05	1.33562	dimer
A19	benzaldehyde	bitter almond, cherry, nutty	1550.1	1395.904	1.15433	
A20	(*E*)-2-nonenal	fatty, green, waxy	1574	1469.796	1.40927	
A21	beta-cyclocitral	fruity, green, mint	1738.4	2096.499	1.31811	
alcohols						
A22	ethanol	aromaticity	945.7	315.319	1.13173	
A23	2-butanol	fruity	1039.9	399.404	1.15475	
A24–1	1-propanol	alcohol, pungent	1054.4	416.474	1.11603	monomer
A24–2	1-propanol	alcohol, pungent	1055.3	417.535	1.24169	dimer
A25–1	2-methyl-1-propanol	fresh, alcoholic, leather	1110.6	493.419	1.17487	monomer
A25–2	2-methyl-1-propanol	fresh, alcoholic, leather	1111.3	494.639	1.37844	dimer
A26	3-pentanol	fusel oil, green	1124.1	516.796	1.20783	
A27	2-pentanol	fusel oil, green	1135.7	537.767	1.22192	
A28	1-butanol	wine	1161.5	587.457	1.18586	
A29–1	1-penten-3-ol	ethereal, green, tropical fruity	1176.9	619.334	0.93091	monomer
A29–2	1-penten-3-ol	ethereal, green, tropical fruity	1176.9	619.334	1.38146	dimer
A30–1	3-methyl-1-butanol	whiskey, banana, fruity	1222.7	694.246	1.24959	monomer
A30–2	3-methyl-1-butanol	whiskey, banana, fruity	1223.1	694.778	1.50453	dimer
A31	(*Z*)-4-heptenal	grass, oil	1257.2	744.719	1.1463	
A32	3-methyl 3-butenol	sweet, fruity	1265.5	757.47	1.17597	
A33–1	1-pentanol	balsamic	1266.3	758.711	1.25391	monomer
A33–2	1-pentanol	balsamic	1266.9	759.595	1.51772	dimer
A34	3-methyl-1-pentanol	wine, cocoa, green, fruity	1311.3	833.218	1.33472	
A35	*cis*-2-penten-1-ol	green, plastic, rubber	1340.4	887.317	0.94031	
A36–1	1-hexanol	fresh, fruity, wine	1371.2	948.295	1.3306	monomer
A36–2	1-hexanol	fresh, fruity, wine	1371.6	949.098	1.64045	dimer
A37–1	(*Z*)-3-hexenol	green, herb	1404.8	1019.704	1.25544	monomer
A37–2	(*Z*)-3-hexenol	green, herb	1405.5	1021.309	1.5086	dimer
A38	2-octanol	fresh, herbaceous, earthy	1440	1100.337	1.44975	
A39	1-octen-3-ol	mushroom, lavender, rose	1487	1217.939	1.16417	
A40	2-ethyl-1-hexanol	citrus, fresh floral, greasy	1539.3	1363.642	1.42677	
A41	2,3-butanediol	sweet, fruity, butter	1691.6	1895.101	1.36566	
ketones						
A42	acetone	fresh, apple, pear	836.6	248.472	1.11325	
A43	2-butanone	fruity, camphor	914.4	294.451	1.24353	
A44	2-pentanone	acetone, fresh, sweet fruity, wine	999.7	355.639	1.35072	
A45	1-penten-3-one	strong pungent odors	1043	403.033	1.08369	
A46	3-hexanone	fruity, grape, sweet, rum	1071.2	437.102	1.18586	
A47	2-hexanone	fruity, fungal, buttery	1099.2	474.479	1.19826	
A48–1	3-penten-2-one	fruity, turns into spicy during storage	1116.2	502.982	1.09135	monomer
A48–2	3-penten-2-one	fruity, turns into spicy during storage	1116.5	503.481	1.34794	dimer
A49	4-heptanone	fruity	1142	549.422	1.23291	
A50–1	2-heptanone	pear, banana, fruity	1193.3	653.868	1.26498	monomer
A50–2	2-heptanone	pear, banana, fruity	1193.3	653.868	1.63091	dimer
A51–1	3-hydroxy-2-butanone	butter, cream	1300.6	814.096	1.09831	monomer
A51–2	3-hydroxy-2-butanone	butter, cream	1300.9	814.676	1.33073	dimer
A52	1-hydroxy-2-propanone	pungent, caramel, fresh	1315.7	841.13	1.09107	
A53	6-methyl-5-hepten-2-one	citrus, fruity, mouldy	1350.4	906.574	1.17633	
A54	2-nonanone	fresh, sweet, green	1398.9	1006.866	1.41366	
A55	2-undecanone	wax, fruity, fat, iris	1692.8	1899.989	1.55491	
A56	3-methyl-2(5H)-furanone	cooked, roasted	1799.1	2390.577	1.1065	
esters						
A57	methyl acetate	ethereal, green, tropical fruity	850.7	256.253	1.19272	
A58	ethyl acetate	fresh, fruity, sweet, grassy	895.3	282.426	1.33131	
A59–1	propyl acetate	fruity, pear	993.5	349.98	1.16869	monomer
A59–2	propyl acetate	fruity, pear	993.5	349.98	1.47638	dimer
A60	isopropyl butyrate	fruit, pungent	1035.6	394.545	1.25832	
A61	methyl 3-methylbutanoate	strong apple, pineapple	1036.9	395.96	1.19734	
A62	ethyl butanoate	pineapple, fruity, whiskey	1054.1	416.12	1.19826	
A63	ethyl acrylate	spicy, pungent	1054.1	416.12	1.40708	
A64	ethyl 2-methylbutanoate	apple, sour and sweet	1068.3	433.451	1.25462	
A65	ethyl 3-methylbutanoate	apple, sour and sweet	1081.6	450.428	1.2768	
A66	butyl acetate	fruity	1088.3	459.27	1.25555	
A67	ethyl pentanoate	apple, pineapple, green	1146.9	558.767	1.25728	
A68	ethyl hexanoate	pineapple, fruity, wine	1250.8	735.156	1.33091	
A69	1-octen-3-one	earthy, mushroom, vegetable	1319.7	848.383	1.26356	
A70	*cis*-3-hexenyl acetate	fresh green grassyy, sweet	1343.5	893.219	1.29733	
A71	ethyl 2-hydroxy-4-methylpentanoate	blackberry, fruity	1691.6	1895.101	1.30384	
others						
A72	dimethyl sulfide	cabbage, sulfur, gasoline	791.4	225.128	0.95803	
A73	2-methylfuran	chocolate, ether like odor	880.7	273.583	0.97373	
A74	acetal	floral, fruity	907.7	290.207	1.0227	
A75	2-ethylfuran	bean, bread, malt, caramel	970.7	333.003	1.0458	
A76	acetonitrile	floral	1031.9	390.301	1.05227	
A77	ethylbenzene	aromatic odor	1146.6	558.236	1.07706	
A78	myrcene	must, spice, balsamic	1162.6	589.708	1.21633	
A79	alpha-terpinene	woody, lemon, citrus	1190.9	649.667	1.22884	
A80	o-xylene	geranium	1191.4	650.681	1.09245	
A81	2-pentylfuran	bean, fruity, earthy, green	1244.8	726.124	1.25069	
A82	2-ethylpyridine	grass	1276.8	775.051	1.10212	
A83	terpinolene	fresh, woody, sweet	1316.4	842.449	1.22616	
A84	2-acetylfuran	fatty, sweet, nutty	1540.8	1368.063	1.12592	

a aroma compounds are determined according to NIST 20 and IMS 23 databases.

b Retention index (RI) calculated on DB-WAX column using n-ketones (C_4_-C_9_).

c Retention time (Rt) in the capillary GC column.

d Drift time (Dt) in the drift tube. FWP, fresh wolfberry pulp. DWP, dried wolfberry pulp. MWP, mixed wolfberry pulp.

## 4 Conclusions

In summary, the aroma compounds in wolfberry pulps were comprehensively identified and characterized. Sensory evaluation revealed that FWP and MWP displayed pronounced fruity, floral, and green aromas, whereas DWP was dominated by sweaty, hay-like, fatty, and herbal notes. Using GC × GC-TOFMS and GC–MS-O analyses, a total of 61 odorants were identified across FWP, DWP, and MWP. Among them, 19, 7, and 16 key aroma-active compounds were confirmed in FWP, DWP, and MWP, respectively, based on FD factors ≥9 and OAVs ≥1. In particular, hexanal, (*E*)-2-hexenal, geranylacetone, 2-undecanone, 6-methyl-5-hepten-2-one, 1-hexanol, 4-hexen-1-ol, 2-hexen-1-ol, 2-pentylfuran, eremophilene, and β-selinene were identified as the key aroma-active compounds in FWP. Additionally, comparative analyses of aroma compound concentrations and OAVs effectively delineated the characteristic aroma markers of FWP. Moreover, HS-GC-IMS generated distinct volatile fingerprints, enabling clear differentiation of 84 odorants among FWP, DWP, and MWP. Overall, investigating the key aroma profiles of fresh, dried, and mixed wolfberry pulps provides a theoretical foundation for establishing aroma-based quality evaluation standards. Meanwhile, identifying these key aroma-active compounds offers valuable guidance for improving the sensory quality and processing techniques of wolfberry pulp products.

## CRediT authorship contribution statement

**Zhifeng Zhang:** Writing – review & editing, Writing – original draft. **Zehao Li:** Software, Methodology, Investigation, Data curation. **Gang Fan:** Supervision, Funding acquisition. **Lulu Zhang:** Writing – review & editing, Supervision, Funding acquisition, Conceptualization. **JingNan Ren:** Writing – review & editing, Investigation. **Kangning Wu:** Writing – review & editing. **Youli Ma:** Writing – review & editing.

## Ethical statement

All participants provided oral informed consent and were fully informed of the experimental requirements and potential risks. Ethical approval for the sensory experiment (HautEC2450) was granted by the Ethics Committee of Henan University of Technology.

## Funding

This study was supported by Yinchuan Science and Technology Plan Project (2024NYHZC006), the Key Research and Development Project of Henan Province (grant number 231111111800, 251111113400), the 10.13039/501100002858China Postdoctoral Science Foundation (grant number 2024 M760795), the Postdoctoral Research Funding Project of Henan Province (grant number HN2025039), Joint Fund Project of Science and Technology R&D Plan of Henan Province (grant number 242103810080).

## Declaration of competing interest

The authors declare that they have no known competing financial interests or personal relationships that could have appeared to influence the work reported in this paper.

## Data Availability

Data will be made available on request.
